# Intergenomic single nucleotide polymorphisms as a tool for bacterial artificial chromosome contig building of homoeologous *Brassica napus* regions

**DOI:** 10.1186/1471-2164-15-560

**Published:** 2014-07-04

**Authors:** Hieu Xuan Cao, Renate Schmidt

**Affiliations:** Leibniz Institute of Plant Genetics and Crop Plant Research (IPK), Corrensstraße 3, OT Gatersleben, D-06466 Stadt Seeland,, Germany

**Keywords:** BAC contig, BAC library, *Brassica napus*, Homoeologous regions, Multidimensional pools, PCR screening, Polyploidy, SNP

## Abstract

**Background:**

Homoeologous sequences pose a particular challenge if bacterial artificial chromosome (BAC) contigs shall be established for specific regions of an allopolyploid genome. Single nucleotide polymorphisms (SNPs) differentiating between homoeologous genomes (intergenomic SNPs) may represent a suitable screening tool for such purposes, since they do not only identify homoeologous sequences but also differentiate between them.

**Results:**

Sequence alignments between *Brassica rapa* (AA) and *Brassica oleracea* (CC) sequences mapping to corresponding regions on chromosomes A1 and C1, respectively were used to identify single nucleotide polymorphisms between the A and C genomes. A large fraction of these polymorphisms was also present in *Brassica napus* (AACC), an allopolyploid species that originated from hybridisation of A and C genome species. Intergenomic SNPs mapping throughout homoeologous chromosome segments spanning approximately one Mbp each were included in Illumina’s GoldenGate® Genotyping Assay and used to screen multidimensional pools of a *Brassica napus* bacterial artificial chromosome library with tenfold genome coverage. Based on the results of 50 SNP assays, a BAC contig for the *Brassica napus* A subgenome was established that spanned the entire region of interest. The C subgenome region was represented in three BAC contigs.

**Conclusions:**

This proof-of-concept study shows that sequence resources of diploid progenitor genomes can be used to deduce intergenomic SNPs suitable for multiplex polymerase chain reaction (PCR)-based screening of multidimensional BAC pools of a polyploid organism. Owing to their high abundance and ease of identification, intergenomic SNPs represent a versatile tool to establish BAC contigs for homoeologous regions of a polyploid genome.

**Electronic supplementary material:**

The online version of this article (doi:10.1186/1471-2164-15-560) contains supplementary material, which is available to authorized users.

## Background

*Brassica napus* (AACC) is an allopolyploid species [1] that most probably originated from inter-specific hybridisation of two *Brassica* species that diverged approximately four million years ago [2], *Brassica rapa* (AA) and *Brassica oleracea* (CC). The genomes of the extant diploid *Brassica* species such as *Brassica rapa*, *Brassica oleracea* and *Brassica nigra* are characterised by a fundamentally triplicated structure [3–6], indicative of a hexaploidisation event in the *Brassica* lineage that post-dated its divergence from the *Arabidopsis* lineage. The triplicated genome regions remained largely collinear although they have been subjected to structural alterations; interspersed gene loss happened particularly often [7–11].

Studies in *Brassica* species greatly benefit from genomic resources that have been assembled. BAC contig maps for the *Brassica rapa* [12] and *Brassica oleracea* genomes were produced [13]. In both studies, high-information-content fingerprinting data of BACs were exploited to establish overlaps between BACs. Integration of the resulting contigs with molecular marker maps was achieved by hybridising sequence-tagged probes to the gridded BAC libraries. For *Brassica oleracea*, almost 600,000 genome survey sequences (GSS) with an average length of 677 bp were established [14, 15] and a draft genome sequence is available for BLAST analyses [16]. In addition, large collections of expressed sequence tags (ESTs) and BAC end sequences were put together for different *Brassica* species [17]. Most importantly, a draft genome sequence was released for *Brassica rapa* [18].

A BAC contig map for the *Brassica napus* genome is not yet available, but *Arabidopsis thaliana* genes were used as probes to screen *Brassica napus* BAC libraries by colony hybridisation in order to identify BAC clones and/or contigs in regions of interest [8, 10, 19–21]. Due to the complex structure of the *Brassica napus* genome it is essential that the BAC clones resulting from such screens are assigned to different loci before they can be characterised in detail. Since a high-quality reference sequence for the *Brassica napus* genome is pending, studies of specific genomic regions at sequence level relied on the analysis of BAC clones and/or contigs. Sequence comparisons between corresponding A genome regions of *Brassica rapa* ssp. *trilocularis* and *Brassica napus* var. Tapidor revealed SNP frequencies varying from 0.82 to 1.98%. Similar values were obtained when C genome copies derived from *Brassica oleracea* ssp. *alboglabra* and *Brassica napus* var. Tapidor were studied [10]. Small differences with respect to gene content and mobile elements were also detected in such comparisons [20]; nevertheless it is clear that studies in *Brassica napus* can draw on sequence resources that have been assembled for the progenitor genomes, *Brassica rapa* and *Brassica oleracea*.

The identification of single nucleotide polymorphisms (SNPs) in *Brassica napus* is hampered by the complex genome structure and high level of sequence identity between A and C subgenome sequences [22]. Due to polyploidy, two classes of polymorphisms need to be considered. Sites that are polymorphic between accessions represent the so-called intragenomic SNPs [23] that are especially versatile for genetic mapping and discrimination of accessions. Large SNP collections have been put together for *Brassica napus* [24–26]. SNPs that differentiate between the homoeologous A and C genomes were termed interhomoeolog polymorphism [24] or intergenomic SNP [23]. This SNP class proved to be valuable for the screening of BAC libraries, since intergenomic SNPs identify and at the same time differentiate homoeologous sequences [21]. Compared to intragenomic SNPs, intergenomic SNPs are much more common [24].

Oligonucleotide extension and ligation assays form the basis for Illumina’s GoldenGate® Genotyping Assay [27–29]. For each SNP locus two allele-specific oligonucleotides that carry the discriminating bases at their 3′-ends are labelled with Cy3 and Cy5, respectively. Additionally, a locus-specific oligonucleotide corresponding to sequences located downstream of the SNP is developed that also carries an Illumicode address sequence. The oligonucleotides are hybridised to the DNA templates to be analysed and a polymerase fills the gap between one of the allele-specific oligonucleotides and the locus-specific oligonucleotide, subsequently a ligase generates a contiguous template. The amplified dye-labelled products are annealed to beads of an optical array which are coated with sequences complementary to the different Illumicode address sequences. Near pure Cy3 or Cy5 fluorescence will be observed for homozygous loci in diploid organisms, in contrast heterozygous loci will elicit both types of fluorescence. Assays which are specific for one of the genomes in an amphidiploid organism will produce Cy3/Cy5 fluorescence ratios indistinguishable from those observed for diploid organisms. In contrast, assays which match target sequences on the homoeologous chromosomes may either segregate on one of the genomes or more rarely on both genomes. In such cases Cy3/Cy5 fluorescence ratios will result that are different from those found in diploid organisms [23, 30]. It is also possible to use Illumina’s GoldenGate® Genotyping Assay for screening of BAC pools [31, 32]. If BAC pools are analysed for the presence of a particular intergenomic SNP with Illumina’s GoldenGate® Genotyping Assay near pure Cy3 or Cy5 fluorescence will be observed for all BAC pools containing only one of the homoeologous loci. In contrast, BAC pools harbouring both loci will elicit both types of fluorescence. Residual fluorescence will result if BAC pools are evaluated that consist solely of BACs that do not contain any of the homoeologous loci [21].

In a previous study a multidimensional screening platform was developed for a *Brassica napus* BAC library. Furthermore, the suitability of Illumina’s GoldenGate® Genotyping Assay for the screening of this library was evaluated. By applying a SNP calling method especially tailored for the analysis of BAC pools it was possible to identify approximately 80% of known BAC coordinates from the BAC library regardless whether intra- or intergenomic SNPs were used. However, it was also recognised that only SNP assays that discriminated between (paleo)homoeologous sequences could be effectively used. Otherwise, even the use of seven or eight screening dimensions did not suffice to identify manageable lists of putative BAC coordinates for the library with tenfold genome coverage. Thus, adequate sequence information is an indispensable prerequisite in order to identify SNPs suitable for BAC library screens [21].

In this study it was tested whether intergenomic SNPs suitable for multiplex PCR screening of *Brassica napus* BAC pools can be effectively deduced by drawing on the ever-increasing sequence resources for the progenitor genomes of *Brassica napus*, *Brassica oleracea* and *Brassica rapa* [16]. Moreover, it was assessed whether intergenomic SNPs can be exploited to establish BAC contig information for homoeologous regions of the *Brassica napus* genome. This proof-of-concept study reveals factors that need to be considered in order to apply the described methodology.

## Results and discussion

### Identification of regions suitable for development of intergenomic SNP assays

**Figure 1:**
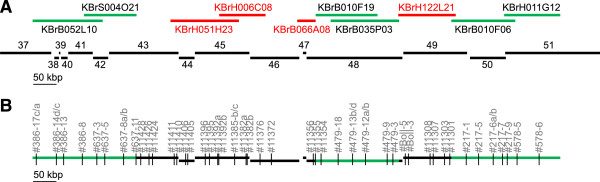
*Brassica rapa* **region on chromosome A1 used for design of intergenomic SNPs. (A)** Positions of BAC sequences relative to WGS sequence contigs. Green lines mark BAC sequences that were used for assay design, whereas red lines represent sequence contigs from working draft sequences of BAC clones that linked the WGS sequence contigs shown as black lines. Designations 37 – 51 refer to accession numbers AENI01000037.1 – AENI01000051.1. **(B)** Position of SNP assay sequences used in this study relative to the region of interest.

A region represented by 15 whole genome sequence (WGS) contigs and spanning approximately one Mbp on *Brassica rapa* chromosome A1 and its counterpart on *Brassica oleracea* chromosome C1 were chosen for this study. Parts of the *Brassica rapa* region were also represented in six completely sequenced *Brassica rapa* BACs (Figure 1A), the *Brassica oleracea* segment included a 45 kbp *Brassica oleracea* ssp. *alboglabra* sequence contig (HG738130).

Intergenomic SNP assays should be placed in regions in which the 20–25 bp of homoeologous sequences flanking the SNP site are identical, moreover they should be distinguished by multiple mismatches and/or indels very close to the assay site when compared to homologous sequences elsewhere in the *Brassica napus* genome [21]. The six *Brassica rapa* BACs were used as BLASTN [33] queries to identify corresponding sequences in *Brassica oleracea* WGS contigs. High scoring segment pairs (HSPs) spanning at least 500 bps and with sequence identities ≥95% were deemed suitable, provided that the hit sequences mapped to the target region on *Brassica oleracea* chromosome C1 and did not reveal additional matches ≥150 bps and with sequence identities of ≥90% outside the region of interest. In total, 59 different HSPs with an average length of 827 bps and an average sequence identity of 96.3% were identified. For the *Brassica oleracea* ssp. *alboglabra* sequence contig corresponding *Brassica rapa* WGS sequences were identified in the same manner. Four HSPs fulfilled all criteria described above; lowering sequence identity to 92% resulted in eight HSPs.

For those regions that were not covered by completely sequenced BACs a different strategy had to be adopted since *Brassica oleracea* O212 WGS sequences were only temporarily available for BLASTN during the time of SNP assay design. Sequences corresponding to annotated *Brassica rapa* genes were extracted and used for megablast searches [34] with the *Brassica oleracea* GSS dataset [14, 15]. HSPs showing ≥90% sequence identity were found for approximately two-thirds of the 98 *Brassica rapa* genes tested. Overlapping GSS sequences were assembled such that only identical segments were retained to minimise the effect of SNPs between the different *Brassica oleracea* lines used for GSS sequencing and of sequencing errors in single pass sequences. In total, 57 different GSS contigs corresponding to 33 different genes were established that showed the highest BLASTN scores for *Brassica rapa* chromosome A1 sequences. The resulting HSPs spanned on average 368 bp and showed a sequence identity of 95.6%.

### Development of intergenomic SNP assays

PCR amplicons were designed based on 29 HSPs such that the homoeologous A and the C genome sequences should be amplified concomitantly in *Brassica napus*. Sequenced amplification products of *Brassica napus* var. Express were inspected for regions suitable for intergenomic SNP assays. For 46 (75.4%) out of 61 potential assay sequences tested the intergenomic SNP identified in *Brassica napus* was also found in the relevant HSP. However, in nine cases the HSPs showed one or more additional sequence polymorphisms close to the assay site so that this particular assay would not have been selected based on the HSP sequences. In conclusion, intergenomic SNPs for the homoeologous regions of the A and C genomes of *Brassica napus* can be inferred from HSPs at reasonable frequency.

Only a subset of HSPs was chosen for assay design. For the entire region 52 assays were developed (Figure 1B, Additional file 1), 18 were solely based on HSP sequences, for another 34 *Brassica napus* amplicon sequences and HSP sequences were analysed.

### Screening of the *Brassica napus*BAC library with intergenomic SNP assays

Table 1 summarises the results of the BAC library screening with Illumina’s GoldenGate® Genotyping Assay. The 52 assays identified between 0 and 3124 putative BACs. In total, 12187 scores corresponding to 8100 different BACs were found. Approximately a quarter of the BACs were identified with two or more assays, more than 550 putative BACs were revealed by four or more assays (Table 2).

**Table 1:** **Summary of BAC library screen**

SNP assay	No of BACs	Assay nucleotide 1		Assay nucleotide 2	
		Identity	No of BACs	Identity	No of BACs
#386-17c	95	C	87	G	8
#386-17a	60	T	51	C	9
#386-14d	2	A	0	G	2
#386-14c	129	A	126	G	3
#386-13	110	A	108	G	2
#386-8	406	C	400	T	6
#637-3	215	T	209	G	6
#637-5	243	C	233	A	10
#637-8a	402	T	336	C	66
#637-8b	214	C	172	T	42
#637-11	538	G	427	A	111
#11428	194	A	142	T	52
#11426	236	A	145	G	91
#11424	3124	T	3124	A	0
#11411	102	T	79	G	23
#11410	80	A	67	T	13
#11406	66	A	58	G	8
#11405	68	G	54	T	14
#11396	22	C	22	G	0
#11395	16	G	11	A	5
#11392a	7	T	7	C	0
#11392b	20	A	10	C	10
#11385-b	21	G	10	A	11
#11385-c	27	G	11	T	16
#11382a	67	T	31	C	36
#11382b	68	G	36	C	32
#11376	406	A	44	T	362
#11372	607	A	607	C	0
#11356	60	A	2	G	58
#11355	182	A	98	G	84
#11354	432	T	82	G	350
#479-18	143	G	65	C	79
#479-13d	60	G	51	A	9
#479-13b	60	A	52	G	8
#479-12b	125	G	121	T	4
#479-12a	123	T	118	C	5
#479-9	152	A	152	G	0
#479-3	286	A	278	G	8
#BolI-5	301	T	297	C	4
#BolI-3	298	C	298	T	0
#11308	377	T	201	C	177
#11307	209	C	118	T	91
#11303	0	G	0	A	0
#11301	1	T	0	C	1
#217-1	117	A	19	G	98
#217-5	741	A	741	G	0
#217-6a	31	C	17	A	14
#217-6b	498	G	488	T	10
#217-7	127	C	74	G	53
#217-9	96	G	64	A	32
#578-5	95	A	63	G	32
#578-6	128	A	105	C	23

**Table 2:** **Many putative BACs were identified by multiple SNP assays**

No of assays	1	2	3	4	5	6	7	8	9	10
No of putative BACs	6051	1106	387	280	122	96	25	20	8	5
No of putative BACs*	3875	912	348	220	127	71	24	16	7	2

*excluding results for assay #11424.

Assays based on intergenomic SNPs are expected to identify with the alternative assay nucleotides two different sets of putative BACs. Such results were observed for 42 assays and classification with respect to the alternative assay nucleotides was unambiguous for 7228 (99.97%) out of 7230 BACs. For nine assays putative BACs were found with only one of the assay nucleotides, but in case of #11301 only a single putative BAC was detected. Assay #11303 did not reveal any putative BAC (Table 1).

### Assignment of assay nucleotides to the A and C genomes

**Figure 2:**
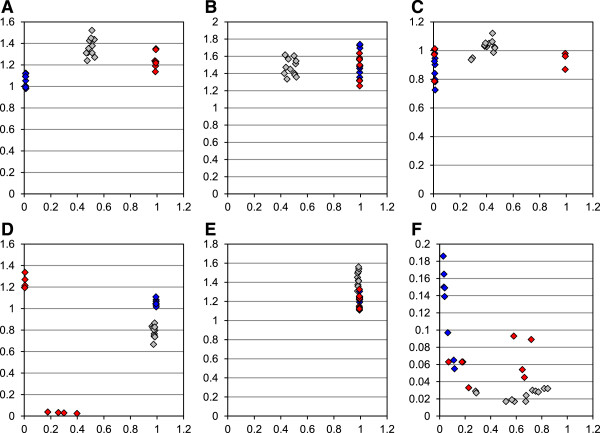
**Analysis of** *Brassica* **accessions with oligonucleotide pool assays.** Normalised fluorescence intensities and normalised Theta values are indicated on the x- and y-axis, respectively. Values for *Brassica napus* var. Express, *Brassica rapa* and *Brassica oleracea* accessions are displayed in grey, red and blue, respectively. For *Brassica rapa* and *Brassica oleracea* two accessions were analysed with four replicates each. Two independent plants with six replicates each were examined for *Brassica napus* var. Express. Panels **A, B** and **C** show the results for assays #637-11, #578-5 and #479-12b, the data for #386-14d, #11396 and #11303 are presented in panels **D, E** and **F**, respectively.

Two different *Brassica oleracea* and *Brassica rapa* accessions each as well as *Brassica napus* var. Express were also analysed with Illumina’s GoldenGate® Genotyping Assay in order to assign the different SNP assay nucleotides experimentally to the A and C genomes. After normalisation, signal intensities for the different samples (normalised R) were plotted relative to allele frequencies (normalised Theta). In these plots, plants homozygous for the alternative assay nucleotides are revealing normalised Theta values close to 0 and 1, respectively. In case of true intergenomic SNPs, *Brassica napus* accessions are expected to show clusters of normalised Theta values clearly distinct from the clusters of *Brassica rapa* and *Brassica oleracea* accessions that are in the majority of cases homozygous for the alternative assay nucleotides (Figure 2A-C). This was observed for 39 (75%) out of the 52 assays tested (Table 3). For eight assays only one of the assay nucleotides revealed a signal with *Brassica napus* var. Express (Table 3, Figure 2D-E). SNP assignment was questionable for *Brassica napus* var. Express for five out of the 52 assays tested (Table 3), among these were two assays, #11303 and #11301, for which very low normalised R values prevented reliable SNP scoring (Figure 2F).

**Table 3:** **Assignments of SNP assay nucleotides to A and C (sub)genomes**

Assay ^(Category)^	*Brassica rapa*			*Brassica oleracea*			*Brassica napus*			
	No1	No2		No1	No2		Express		BAC A	BAC C
	SNP	SNP	WGS	SNP	SNP	WGS (GSS)	SNP	Sequences of PCR amplicons		
#386-17c^(1)^	C	C	C	G	G	G	C/G	S	C	G
#386-17a^(1)^	T	T	T	C	C	C	T/C	Y	T	C
#386-14d^(4)^	A	-	A	G	G	G	G	S	C	G
#386-14c^(1)^	A	A	A	G	G	G	A/G	R	A	G
#386-13^(1)^	A	A	A	G	G	G	A/G	R	A	G
#386-8^(2)^	C	C	C	T	C	T	T/C	Y	C	T
#637-3^(1)^	T	T	T	G	G	G	T/G	K	T	G
#637-5^(2)^	A/C	C	A	A	A	A	A/C	M	C	A
#637-8a^(1)^	T	T	T	C	C	C	T/C	Y	T	C
#637-8b^(1)^	C	C	C	T	T	T	T/C	Y	C	T
#637-11^(1)^	G	G	G	A	A	A	A/G	R	G	A
#11428^(1)^	A	A	A	T	T	T (T)	T/A	W	A	T
#11426^(1)^	A	A	A	G	G	G (G)	A/G	R	A	G
#11424^(5)^	A	A	A	T	T	T (T)	T	T	T	T
#11411^(3)^	T/G	T	T	G	T	T (T)	T/G	K	T	G
#11410^(2)^	A	A	A	A	A	A (T)	A/T	W	A	T
#11406^(3)^	A	A	A	A	A	A (A)	A/G	R	A	G
#11405^(1)^	G	G	G	T	T	T (T)	T/G	K	G	T
#11396^(5)^	C	C	C	C	C	C (G)	C	C	C	C
#11395^(1)^	G	G	G	A	A	A (A)	A/G	R	G	A
#11392a^(4)^	T	T	T	C	-	T (C)	T	T	T	T
#11392b^(2)^	A	A	A	C	C	A (C)	A/C	M	A	C
#11385-b^(1)^	G	G	G	A	A	A (A)	A/G	R	G	A
#11385-c^(1)^	G	G	G	T	T	T (T)	T/G	K	G	T
#11382a^(1)^	T	T	T	C	C	C (C)	T/C	nd	T	C
#11382b^(2)^	G	G	G	G	C	C (G)	G/C	S	G	C
#11376^(1)^	A	A	A	T	T	T (T)	T/A	W	A	T
#11372^(5)^	C	A	C	A	A	A (A)	A	nd	A	A
#11356^(3)^	nd	-	A	G	G	A (G)	A/G	nd	A	G
#11355^(1)^	A	A	A	G	G	G (G)	A/G	R	A	G
#11354^(1)^	T	T	T	G	G	G (G)	T/G	K	T	G
#479-18^(1)^	G	G	G	C	C	C	nd	S	G	C
#479-13d^(2)^	G	A/G	A	A	A	A	A/G	R	G	A
#479-13b^(2)^	A	A/G	A	G	G	G	A/G	R	A	G
#479-12b^(2)^	G	T	G	T	T	T	T/G	K	G	T
#479-12a^(3)^	T	T	T	T	T	T	T/C	Y	T	C
#479-9^(4)^	nd	G	G	A	A	A	A	A	A	nd
#479-3^(2)^	A	G	G	-	G	G	A/G	R	A	G
#BolI-5^(2)^	T/C	T	T	C	C	C	nd	T	T	C
#BolI-3^(4)^	C	C	C	T	T	T	C	C	C	nd
#11308^(1)^	T	T	T	C	C	C (C)	T/C	Y	T	C
#11307^(2)^	C	C	C	T	C	T (C)	T/C	Y	C	T
#11303	nd	nd	G	nd	nd	A (A)	nd	nd	G	G
#11301	nd	nd	T	nd	nd	C (C)	nd	Y	T	C
#217-1^(1)^	A	A	A	G	G	G	A/G	R	A	G
#217-5^(5)^	G	G	G	A	A	A	A	nd	A	A
#217-6a^(3)^	A	A	A	A	A	A	A/C	M	C	A
#217-6b^(2)^	G	G	G	nd	nd	T	nd	K	G	T
#217-7^(2)^	C	C	G	G	G	G	C/G	S	C	G
#217-9^(1)^	G	G	G	A	A	A	A/G	R	G	A
#578-5^(3)^	G	G	G	G	G	G	A/G	R	A	G
#578-6^(1)^	A	A	A	C	C	C	A/C	C	nd	C

Assay categories are described in detail in the Results section. No1 and No2 refer to the *Brassica rapa* and *Brassica oleracea* accessions that were analysed by using Illumina’s GoldenGate® Genotyping assay. For *Brassica napus* var. Express, plant DNA samples and BACs that had been allocated to the A and C subgenomes were examined. The columns designated “SNP” show the SNP assay nucleotides that were found by using Illumina’s GoldenGate® Genotyping assay. Which of the different assay nucleotides matched the whole genome shotgun sequences (WGS) and/or genome survey sequences (GSS) is displayed in the columns “WGS” and “WGS (GSS)”. Data points that were not determined are marked as “nd”.

The two *Brassica rapa* accessions were assigned to the same SNP with 39 assays (Table 3, Figure 2A, 2B, 2E), for the two *Brassica oleracea* accessions this was observed for 43 assays (Table 3, Figure 2A-B, 2D-E). Cases in which individual *Brassica rapa* and/or *Brassica oleracea* accessions carry different alleles and/or genes were also observed (Figure 2C). In case of assay #386-14d one of the *Brassica rapa* accessions revealed residual fluorescence, thus it is either missing corresponding sequences or carries an allele that cannot be detected with this particular assay (Figure 2D).

Sequence alignments of the assay sequences to the GSS sequences of *Brassica oleracea* TO1000DH3 or TO1434 [14, 15] as well as WGS sequences of *Brassica rapa* Chiifu-401 [18] and *Brassica oleracea* O212 were also exploited to determine which of the assay nucleotides corresponded to the A and C genomes, respectively. The analysis of 24 assay sequences for which WGS and GSS contig sequences were available revealed in the majority of cases the same progenitor genome assignment, differences were noted in seven cases (Table 3).

PCR amplicons aimed at the amplification of the homoeologous regions (Additional file 2) were used to establish sequences for *Brassica napus* var. Express across all assay sites. At 39 SNP assay sites *Brassica napus* var. Express amplicon sequences revealed both assay nucleotides, but for #386-14d C and G were found instead of the assay nucleotides A and G. For seven SNP sites only one of the assay nucleotides was observed in the amplicon sequences, in five of these cases the results were consistent with the SNP genotyping of *Brassica napus* var. Express. In case of #578-6, an intergenomic SNP was observed by SNP genotyping whereas the amplicon sequence was specific for the C genome copy. Indels differentiating the homoeologous sequences rendered the *Brassica napus* var. Express amplicon sequences non-readable across the assay sites in five cases (Table 3).

### Assignment of putative BACs to the A and C subgenomes

For each of the markers bioinformatic and experimental assignments of the assay nucleotides to the *Brassica rapa* and *Brassica oleracea* genomes were assessed in order to allocate tentatively the putative BACs to the A and C subgenomes. At first, the 42 assays were evaluated for which both assay nucleotides had identified sets of BACs (Table 1). In case of 23 assays one of the assay nucleotides was consistently classified as *Brassica oleracea* and the other as *Brassica rapa* (Table 3, assay category 1). All BACs detected by these assays were assigned to the corresponding subgenomes. For a set of thirteen assays inconsistencies were found for one of the assay nucleotides, but concordant assignments for the other assay nucleotide (Table 3, assay category 2) permitted to ascribe the putative BACs to the appropriate subgenomes. Thus, for 36 out of 42 assays which revealed the pattern for an intergenomic SNP with *Brassica napus* var. Express, it was possible to assign the assay nucleotides to the A and C subgenomes based on the data established for the progenitor genomes. In the remaining six cases (Table 3, assay category 3) the classification of BACs that had been identified with markers of categories 1 and 2 was used to infer the subgenome assignments for the different assay nucleotides.

Eight assays had revealed signals with only one of the assay nucleotides for DNA samples of *Brassica napus* var. Express. Such a pattern may result if one of the subgenomes carries one or several sequence polymorphisms that interfere with binding of the assay oligonucleotides. In such a case all BACs identified will belong to the same subgenome, therefore it is possible to infer the subgenome assignment with the help of markers that allowed for unambiguous assignment (Table 3, assay category 4). In contrast, subgenome assignments were not made in cases in which the A and C subgenome copies of *Brassica napus* var. Express are identical in sequence in the assayed region, since such assays identified two different sets of putative BACs that were predicted by other assays to represent the A and C subgenomes, respectively (Table 3, assay category 5).

Subgenome assignments of the assay nucleotides corresponding to assays #11303 and #11301 were based on the sequence comparison with WGS sequences only, since poor fluorescence intensity values prevented a meaningful evaluation of the genotyping results (Table 1).

**Figure 3:**
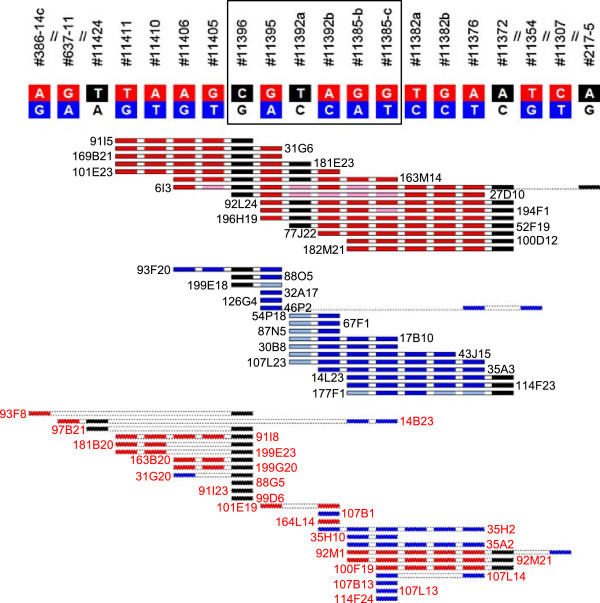
**Subgenome assignment of BACs.** All 57 putative BACs that were identified with the six SNP assays surrounded by the box are shown as rectangles. SNP assays were ordered according to their sequence in the *Brassica rapa* genome. Two slashes indicate that one or more of the SNP assays used in this study map in the *Brassica rapa* genome between the ones shown. PCR experiments revealed which of the putative BACs truly map to the regions of interest. Black font marks confirmed BACs, whereas BAC coordinates that were not verified to map to the homoeologous regions of interest are shown in red font. Red, dark blue and black boxes indicate SNP scores that were confirmed by PCR analyses, whereas dotted outlines mark false positive SNP scores. Pink or pale blue boxes correspond to scores that were only revealed by PCR but not by Illumina’s GoldenGate® Genotyping Assay. Red or pink colour represent the A subgenome, C subgenome sequences are displayed in dark or pale blue. Scores of assays for which only one of the assay nucleotides revealed putative BACs are shown as black boxes.

Out of the putative 1586 BACs that were identified by two or more markers belonging to assay categories 1 to 4 only 60 (3.8%) showed inconsistencies with respect to subgenome assignment. For markers of categories 1 and 2 a value of 4.2% was found, thus, integrating the scores of categories 3 and 4 did not result in an increased proportion of BACs with conflicting subgenome assignment.Figure 3 shows subgenome assignments for all 57 putative BACs that were identified with a set of six assays (Figure 3). The four assays for which both assay nucleotides were unambiguously ascribed to the A and C subgenomes (#11395, #11392b, #11385-b and #11385-c) detected in total 44 BACs, 35 of which were identified by two or more other assays belonging to categories 1 or 2. Conflicting assignments were observed for only two putative BACs, 14B23 and 92M1, both of which were not truly mapping to the regions of interest.

**Figure 4:**
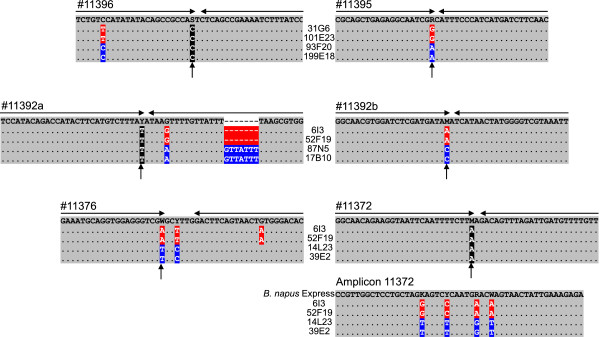
**Analysis of BACs with amplicons spanning SNP assays.** Sequences of PCR amplicons established for representative BACs are shown below six different SNP assay sequences. Horizontal arrows represent the extent of the assay nucleotides. Vertical arrows mark the positions of the SNP assay nucleotides. Differences between A and C subgenome BACs are highlighted in red and blue, respectively. BAC sequences spanning #11396, #11392a and #11372 are monomorphic at the site of the assay nucleotides, nonetheless A and C subgenome BACs can be distinguished based on sequence differences. For amplicon 11372 the region corresponding to the assay is shown and a region mapping approximately 300 bp apart. For the latter the BAC sequences are shown underneath the amplicon sequence of *Brassica napus* var. Express.

For assays #11396, #11392a and #11372 only one of the assay nucleotides each revealed signals with *Brassica napus* var. Express and the BAC pools. BACs identified by assay #11392a had been consistently allocated to the A subgenome based on the flanking markers #11395 and #11392b (Figure 3). Analysis of the C genome BACs that had been found with #11395 and #11392b with PCR amplicons identified seven BACs that harboured sequences matching to #11392a as well as #11392b (Figure 3). Assay #11392a was not suitable to detect BACs carrying the C subgenome sequences because an SNP and a seven-bp indel were found in the region of the C subgenome where assay oligonucleotide 3 should bind (Figure 4). Assays #11396 and #11372 identified BACs that had been allocated to the A and C subgenomes by closely-linked markers (Figure 3). The BACs representing the A and C subgenomes were identical in sequence at the site of the assay nucleotide in both cases, but at other positions sequence differences differentiated the subgenomes (Figure 4). Without exception, the data derived from BAC amplicon sequencing and from SNP scores of flanking markers were consistent.

### Selection of BACs for contig building

For the BAC library an average insert size of 135 to 150 kbp had been estimated [21]. The distance between the selected SNP assays that were directly adjacent to each other in the *Brassica rapa* genome varied from approximately 100 bp to 81 kbp, the median distance was 20 kbp. For SNP assays that were not directly adjacent to each other but which were separated by one or two other markers the median values increased to 37 kbp and 59 kbp, respectively. The maximum distance between assays which were separated by two additional markers amounted to 134 kbp in the *Brassica rapa* genome.

In order to select BACs for validation studies it was reasoned that clones that are truly present in the homoeologous regions of interest should most probably be identified by four or more assays. The results for assay #11424 were not considered in the selection procedure since it had revealed more than 3000 putative BACs. In total, 467 BACs were identified by four or more assays (Table 2). Notably, all of the BACs shown in Figure 3 and that are truly mapping to the A subgenome copy met this condition. Previously it was noted that not all assays are equally well suited to identify BAC clones in the BAC library [21]. Similarly, the data for assays #11392a (Figure 3) and #386-14d in this study had also revealed that individual loci had escaped detection in the library screen. In order to allow for such shortcomings of the screening procedure and/or putative small rearrangements in the *Brassica napus* A and C subgenome copies relative to the progenitor genomes putative BACs were also considered for further analysis if they were identified by any two markers in the region of interest that were separated in the *Brassica rapa* genome by at least two other markers. BACs 46P2 and 177F1 for example are truly mapping to one of the homoeologous regions of interest, despite the fact that they were only identified by three SNP assays (Figure 3). Among all BACs that had not been selected based on the first criterion this condition was met by 232 putative BACs. In addition, the putative BAC coordinates that were identified with the two or three markers directly adjacent to each other and mapping to the very ends of the region of interest in *Brassica rapa* were also taken into account for further studies.

In total, 738 (9.1%) out of the 8100 putative coordinates that had been identified in the screen were selected. All but one of these BAC clones could be recovered from the library and were subjected to validation studies.

### BAC contigs corresponding to homoeologous regions of interest

Analysis with PCR amplicons (Additional file 2) revealed that 114 and 83 BACs corresponding to the A and C subgenomes, respectively, were truly present in the homoeologous regions of interest. All assay sequences which were verified to be present in a particular BAC showed consistent results regarding subgenome assignment.

Out of the 1055 SNP scores that had been obtained for the 197 confirmed BACs only 37 were not verified by analysis with PCR amplicons. Due to its C-genome specificity PCR amplicon A578-6 was not suitable to confirm twelve scores assigned to the A genome with SNP assay #578-6 (Table 3), thus only 25 (2.5%) scores represented false positives.

**Figure 5:**
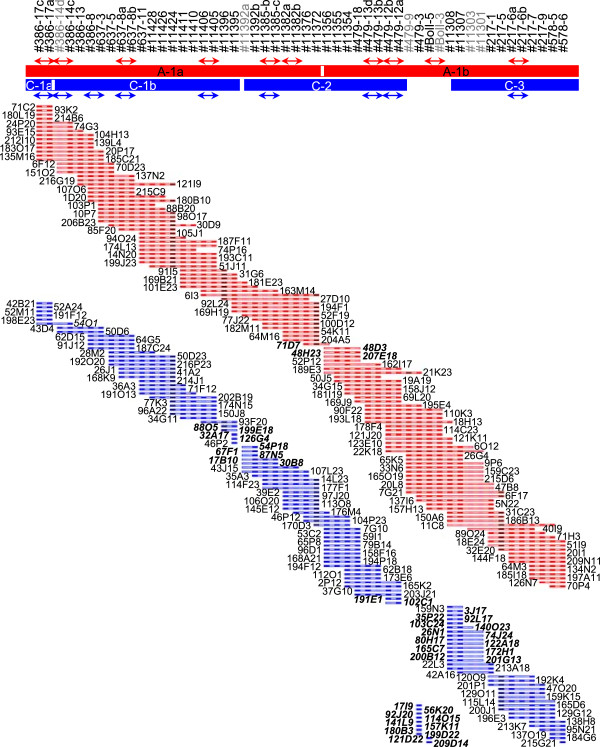
**BAC contigs of homoeologous regions of interest.** The order of 52 SNP assays as found in the *Brassica rapa* genome is indicated at the top. Assays labelled in grey font indicate that a particular assay and/or assay nucleotide did not reveal any BAC that was confirmed to carry sequences corresponding to this assay. BACs that were confirmed to carry sequences corresponding to the SNP assays are displayed as rectangles. Red and dark blue boxes stand for SNP scores that were validated by analyses with PCR amplicons. Pink or pale blue boxes represent false negative SNP scores. Scores corresponding to the A subgenome are shown in red or pink, dark or pale blue boxes are indicative of the C subgenome. Coordinates that belong to the 738 BACs that had been selected initially for the validation studies are labelled in plain black font. BAC contigs are shown as filled rectangles below the names of the assays. Arrows indicate that assay order cannot be determined based on BAC contig data. Coordinates labelled in bold and italics highlight additional BACs that had been integrated at the end of the BAC contigs for the refinements of the contigs. Clear rectangles indicate that BAC contigs were bridged by such additional BACs.

The A and C subgenome BACs were assembled into two and four contigs, respectively (Figure 5). The deduced order of assays in the A subgenome was generally consistent with their arrangement in the *Brassica rapa* genome. However, 20 of the BACs either suggested altered marker arrangement when compared to the *Brassica rapa* progenitor genome and/or their assay scores were not consistent with the order of SNP assays as established by other BACs. For example, BAC 27D10 was not identified by any of the four assays mapping between #11395 and #11382a, although the data for several other BACs suggest a similar order as found in the *Brassica rapa* genome (Figure 3).

In order to refine the contig data, the same marker order as observed in *Brassica rapa* was used to order the BACs, then the extent of all BAC clones was established by analysing amplicons that spanned assays directly adjacent to the assays that had shown a score for a particular BAC. Furthermore, amplicons corresponding to assays that had not yielded a signal in the BAC library screen but which were predicted to be present in the BAC based on the contig data were evaluated. In total, 114 false negative scores were identified, about half of which were attributed to just four markers, #11303, #11301, #386-14d and #11392a. Assays #11303 and #11301 yielded poor fluorescence values; therefore a high rate of false negative scores was expected. The false negative scores for #386-14d and #11392a were caused because these assays were only suitable to detect BACs representing one of the subgenomes (Table 3, Figures 3 and 4). In the remaining 56 cases of false negative scores BACs were not identified because one or several of the BAC pools did not yield a signal above the applied threshold or a BAC pool had been ascribed to the wrong assay nucleotide. The determination of a BAC coordinate required a score in each of seven different screening dimensions, therefore the observed rate of false negative scores has to be regarded as low. These results are in accordance with a previous study [21].Elimination of false positive scores and integration of false negative SNP scores resolved all conflicts with respect to marker order, only the order of assays mapping in very close proximity to each other remained ambiguous (Figure 1B, Figure 5). For selected BACs the progenitor genome assignment was also checked. Without exception, the subgenome assignments were verified.

### Linking of BAC contigs

The criteria which were used to select putative BACs for contig building may not be optimal throughout the entire homoeologous regions of interest, due to uneven spacing of assays (Figure 1B). Therefore, additional putative BACs which had been identified by assays mapping to the very ends of contigs were analysed in order to evaluate whether some of them may link adjacent contigs. The gap between contigs A-1a and A-1b was bridged by BAC 71D7 (Figure 5), likewise BAC 54O1 linked contigs C-1a and C1-b (Figure 5).

In contrast, it was not possible to find BACs that spanned the region between contigs C-1 and C-2 in the C subgenome (Figure 3). The analysis of putative BACs that had been found with assays that were flanking the gap between contigs C-2 and C-3 showed that 2 and 13 additional BACs, respectively were truly mapping to the ends of these contigs, but none of these BACs bridged the gap (Figure 5). In the corresponding region of the *Brassica rapa* genome and the *Brassica napus* A subgenome four markers are found, #479-9, #479-3, #BolI-5 and #BolI-3 (Figure 5). With #BolI-5 three BACs had been ascribed to the C subgenome, two of which were confirmed to contain sequences corresponding to this assay (Figure 5). However, the amplicon sequences established for these two BACs perfectly matched a region of *Brassica oleracea* chromosome 1 which is very similar but not identical to the region of interest (data not shown). With #479-9 eight BACs were ascribed to the C genome. All of them were confirmed to carry sequences corresponding to the assay, but none of these BACs had been identified with any of the other 51 SNP assays used in this study (Figure 5). None of the BACs revealed by assays #479-3 and #BolI-3 had been ascribed to the C genome, moreover, the amplicon sequences for *Brassica napus* var. Express only revealed the assay nucleotide corresponding to the A subgenome. All these data together suggest that this particular region is largely missing from the *Brassica napus* var. Express C subgenome and/or the BAC library. Alternatively, sequence divergence may explain why neither amplicon sequences nor assays were suitable to detect BACs representing the C genome copy.

### Considerations for SNP assay design aiming at contig building of homoeologous regions

Genome assemblies solely relying on short-read sequences are especially challenging in highly heterozygous and polyploid genomes. Strategies aimed at reducing genome complexity are therefore used to alleviate genome assembly problems. These include inbreeding, reduction of ploidy, physical separation of chromosomes and the sequencing of diploid progenitor genomes [35, 36]. Sequencing of diploid progenitor genomes as surrogates for polyploid species has not only been undertaken in the *Brassica* genus, but for example also in potato [37] and *Gossypium* [38]. Due to such efforts it would be possible to apply the methodology described here also in other species. However, it will be important to consider gene density and degree of sequence identity in homoeologous segments to be analysed because these factors will determine whether HSPs suitable for SNP assay design can be identified in adequate number.

Availability of WGS sequence contigs for both progenitor genomes allows the efficient identification of HSPs suitable for the design of intergenomic SNPs. Using the same criteria that had been applied in this study for the regions covered by completely sequenced BACs, HSPs were found for example on average every 9.6 kbp when the *Brassica rapa* WGS sequence contigs AENI01000037.1 – AENI01000051.1 were taken as queries to detect corresponding sequences in the *Brassica oleracea* WGS data set (data not shown). Moreover, the availability of WGS sequence contigs for both progenitor genomes allows elimination of all assay sequences showing highly similar matches in regions other than the target areas. The analysis of the *Brassica oleracea* WGS sequences at the time of assay design would for example have resulted in the omission of assay #BolI-5 that matches two highly similar regions on *Brassica oleracea* chromosome C1. Nonetheless, it is not essential to have full access to both progenitor genome sequences in order to deduce intergenomic SNPs for the study of an allopolyploid genome, as a *Brassica oleracea* GSS data set with 0.6-fold estimated genome coverage [15] also yielded a sufficient number of sequence alignments suitable for the design of intergenomic SNPs in the regions of interest. Genomic survey sequences can be generated in a particularly efficient manner by next generation sequencing technologies, as has recently been demonstrated in the *Brassica* genus for *Brassica juncea* [39]. The establishment of a GSS data set for *Brassica nigra* (BB) would enable the analysis of homoeologous regions in the *Brassica juncea* (AABB) and *Brassica carinata* (BBCC) genomes with the methodology described here.

Comparison of the *Brassica napus* var. Express sequences with the corresponding sequences in the progenitor genomes revealed many instances in which intergenomic SNPs were conserved. Such sites are ideally suited for a BAC library screen. Cases in which an intergenomic SNP was detected in the progenitor genomes but not in *Brassica napus* were also found. Although such assays did not discriminate between homoeologous BACs at least they identified the BAC coordinates containing the loci of interest. However, simultaneous detection of homoeologous sequences by a single assay nucleotide usually leads to a very high number of putative BACs [21]; this was also observed for assays #11424, #11372 and #Ac217-5 in this study (Table 1).

The two types of sequence polymorphisms that will result in detection failures of certain loci were rare. In one case the intergenomic SNP found in *Brassica napus* var. Express differed from the one detected in the progenitor genomes (#386-14d, Table 3). Sequences corresponding to two assays carried in one of the subgenomes additional SNPs and/or Indels in close proximity to the SNP site that interfered with proper functioning of the SNP assay, in case of #11392a none of the BACs containing the locus of the C genome were detected (Figures 3 and 4), for #11356 the identification of A subgenome BACs was not abolished but comparatively poor.

### Strategy for BAC contig building of homoeologous regions

Fingerprinting experiments [40] and whole genome profiling [41] are suitable strategies to build genome-wide BAC contig maps. However, these techniques alone are not suitable to build maps for specific regions of interest. In contrast, map-based physical-mapping approaches allow the generation of clone contig maps for specific regions of interest. Using molecular markers mapping to the region of interest as hybridisation probes will identify and anchor artificial chromosome clones on the genetic map. However, in case of polyploid genomes substantial additional efforts are needed because hybridisation probes will usually not discriminate between (paleo)homoeologous sequences [8, 10, 19–21]. Thus, all identified clones need to be assigned to individual loci before placing them into contigs.

Strategies aimed at multiplex PCR-based screening of multidimensional BAC pools have to consider that for libraries encompassing several genome equivalents usually many putative clones will be detected. Consequently, substantial work has to be invested in order to identify those BACs that are truly present in the region of interest. Considerable less validation effort is necessary if sub-pools with one to twofold genome coverage are used [31], however, studies aiming at contig formation usually employ libraries encompassing at least five to ten genome equivalents, otherwise too many loci will not be detected and/or it will not be possible to determine the order of markers in the contigs. For the BAC library studied here which covers approximately ten genome equivalents it had been shown that out of 24 loci tested one was not found in the library and for another one only a single BAC was detected [21].

As shown in this study the efficiency of map-based physical-mapping approaches can be fully exploited in polyploid genomes if intergenomic SNPs are used for the assembly of homoeologous regions, provided that the intergenomic assays are spaced such that each BAC spans several assays. Then, BACs spanning three or more assays can be effectively selected from the entire set of putative BACs for validation studies. In the course of this study 236 BACs had been placed into contigs in the homoeologous regions of interest, 170 (72%) of which had been selected because they spanned four or more assays. Importantly, this selection procedure even worked well when results of several assays were integrated that did not differentiate between homoeologous sequences and therefore identified several hundred BACs each. Only an assay that detected more than 3000 putative BACs had to be omitted from the analysis (Table 2). Even the results of assays that match homologous sequences outside the regions of interest in addition to the target sequences can be integrated, since the BACs that correspond to such homologous sequences will usually not be identified by other assays in the regions of interest.

In contrast, only a small fraction of putative BACs that had been identified by two or three markers that were separated in the *Brassica rapa* genome by at least two other markers were truly present in the homoeologous regions of interest. Out of the 232 BACs that were selected based on this criterion, only 16 (6.9%) contained sequences mapping to the regions of interest. Instead of choosing BACs based on this criterion it was more fruitful to select BACs that were identified by two or three assays mapping to the ends of contigs. For example, out of the 39 BACs that were mapping to the very ends of the regions of interest 11 (28.2%) were confirmed to be part of the contigs. Moreover, following such a strategy BACs were identified that bridged contigs A-1a and A-1b as well as C-1a and C-1b.

In the study presented here, all SNP scores were validated in order to assess the entire procedure. Taking into account that selection criteria efficiently reduced the number of putative BACs to be tested from more than 8000 to approximately 500, fingerprinting experiments [40] are the method of choice to place the BACs into contigs. In this way contig formation is validated by an independent method, moreover, the extent of BACs relative to each other is established more precisely. This is especially important if minimal tiling paths shall be chosen for sequencing. BAC pools can be established based on the minimal tiling paths and subjected to next generation sequencing technologies as has recently been reported for large homoeologous regions of Upland cotton [42].

A comparison of two *Oryza sativa* reference sequence assemblies revealed that the sequence that was established by a WGS strategy showed many more assembly errors than the sequence that was generated by a clone-by-clone sequencing approach [43]. This emphasises that in all cases in which the exact order of sequence contigs on chromosomes needs to be known it may be necessary to validate assemblies. This will be especially important in cases of highly heterozygous or polyploid genomes that show a high degree of sequence identity in their subgenomes. In such cases validation studies may benefit from the approach described in this study.

## Conclusions

Clone contig maps for genomes can be efficiently generated by BAC fingerprinting, but the assembly of contigs for specific chromosome regions in medium or high-throughput fashion remains a challenge, particularly in polyploid genomes in which highly similar regions need to be discriminated. The screening of multidimensional BAC pools with highly parallel SNP assays has been established, however, for libraries encompassing several genome equivalents considerable additional work has to be invested to identify among the putative BACs revealed in the screen those that are truly corresponding to the assay sequences. These limitations are overcome if a screening procedure is implemented in which intergenomic SNP assays are spaced throughout homoeologous regions of interest such that any BAC truly mapping in the homoeologous regions of interest will be identified by several assays. Taking into account that suitable intergenomic SNPs can be deduced based on WGS sequence resources of diploid progenitor genomes, the proposed approach enables the efficient assembly of BAC contigs for homoeologous chromosome regions of a polyploid genome.

## Methods

### Identification of regions suitable for the detection of intergenomic SNPs

*Brassica rapa* Chiifu-401 WGS (AENI01000037.1 - AENI01000051.1) and BAC sequences (KBrB052L10 - AC189386, KBrS004O21 - AC189637, KBrB010F19 - AC189219, KBrB035P03 - AC232479, KBrB010F06 - AC189217, KBrH011G12 - AC189578) were retrieved from the relevant databases [16, 44]. *Brassica rapa* BAC sequences and a 44.6 kbp segment of *Brassica oleracea* ssp. *alboglabra* (HG738130) were used as queries to identify corresponding WGS sequences of *Brassica oleracea* O212 and *Brassica rapa* Chiifu-401 sequences, respectively [16] with the following BLASTN [33] parameters; nucleotide match 1, nucleotide mismatch -3, gap open penalty 5, gap extension penalty 2, the low complexity filter was applied. As threshold for reporting a match 0.00001 was chosen.

*Brassica rapa* sequences spanning annotated open reading frames and between 100 to 200 bp upstream of the ATG and downstream of the stop codon [16] were used as queries to identify corresponding *Brassica oleracea* GSS sequences with megablast [34] (nucleotide match 1, nucleotide mismatch -2, linear gap costs, no low complexity filter, threshold for reporting a match 0.00001). In cases in which open reading frames mapped in very close proximity to each other (<700 bp), the query sequence spanned both genes and the intergenic region. GSS contigs were established by retaining identical sequences of partially overlapping GSS sequences and used as BLASTN [33] queries with the WGS sequence contigs of the *Brassica rapa* genome [16] applying the same parameters as stated above.

### Amplicon and SNP assay design

BLASTN [33] alignments of *Brassica rapa* and *Brassica oleracea* sequences were used to develop PCR amplicons suitable for the concurrent amplification of homoeologous regions of the *Brassica napus* genome. Primers with a melting temperature of 60°C were selected with Primer3 [45].

BLASTN [33] sequence alignments of *Brassica rapa* and *Brassica oleracea* sequences and/or *Brassica napus* var. Express amplicon sequences of homoeologous regions were inspected for the presence of intergenomic SNPs. If oligonucleotides flanking an intergenomic SNP could be chosen such that they completely matched at least 20–25 bp of the *Brassica napus* sequence the region was deemed appropriate for SNP assay design. Single mismatches were allowed, but only if they were found more than 15 bps away from the SNP site. Regions spanning 60 bps either side of the SNP were extracted. Only assays with a designability score (Illumina Inc. San Diego, CA) higher than 0.6 were considered. In case other homologous sequences had been identified in the progenitor genomes it was taken care that they were differentiated from the assay sequences by mismatches and/or indels as close to the assay site as possible.

### PCR conditions

Standard molecular biology techniques were performed as described [46]. Oligonucleotides were purchased from Eurofins MWG Operon (Ebersberg, Germany) and sequencing was carried out at the IPK Gatersleben (Germany).

PCR amplifications were carried out in reaction volumes of 30 μl that contained 1 × DreamTaq™ buffer, 250 μM dNTP, 30 pmol of each primer and 1 U of DreamTaq® DNA Polymerase (Fermentas, St. Leon Rot, Germany). Twenty ng of *Brassica napus* var. Express template DNA were used for PCR amplification. Single BAC clones were assayed for the presence or absence of a particular amplicon by transferring single bacterial colonies to PCR tubes containing all necessary reagents.

PCR samples were preheated for ten min at 95°C and then subjected to 35 PCR cycles. Each cycle consisted of three steps; 30 s at 94°C, 30 s at 60°C, and 60 s at 72°C. After incubation for five min at 72°C the reactions were cooled down to 15°C. Five-μl aliquots were resolved on agarose gels in 1 × TBE.

Prior to sequencing five-μl aliquots of the amplification products were treated with Exonuclease I and FastAP™ Thermosensitive Alkaline Phosphatase as described by the manufacturer (Fermentas, St. Leon Rot, Germany).

### Analysis with Illumina’s GoldenGate® Genotyping Assay

Sequences containing suitable SNPs were extracted and supplied to Illumina Inc. (San Diego, CA) for design of multiplex oligonucleotide pool assays (OPA, Additional file 1).

DNAs of 276 six-dimensional and 216 single-plate BAC pools [21] were assayed with Illumina’s GoldenGate® Genotyping Assay alongside the DNAs of *Brassica napus* var. Express, two *Brassica oleracea* accessions and two *Brassica rapa* accessions. Plant DNA samples had been amplified with the Illustra GenomiPhi V2 DNA Amplification Kit (GE Healthcare Europe GmbH, Munich, Germany) prior to genotyping. For each of the 276 six-dimensional and the 216 single-plate BAC pools 125 ng and ten ng of template DNA served as input, respectively, for plant DNA samples 250 ng were used.

All genotyping experiments were carried out as recommended by the manufacturer. Fluorescence signals were recorded with a BeadXpress Reader (Illumina Inc. San Diego, CA). Assignment of SNPs to the different BAC pools followed the scheme as described [21]. In brief, only BAC pools showing normalised fluorescence intensities (Normalised R) ≥0.2 were evaluated. BAC pools with normalised Theta values (Normalised Theta) ≤0.05 and ≥0.95 were classified to contain the alternative SNPs, whereas all values found in the range between 0.05 and 0.95 were assigned to the category containing both SNPs.

A Microsoft Excel spreadsheet (Microsoft Corporation, Redmond, USA) in which the pool information for all 82,944 BAC coordinates had been entered provided the basis for clone deconvolution. The filter function was used to enter all pools that had been identified with a particular SNP in order to compile the list of all putative BAC coordinates for each assay nucleotide.

### Analysis of putative BAC clones and contig building

The coordinates of all putative BAC clones that were identified in the BAC library screen were entered into lines of a Microsoft Excel (Microsoft Corporation, Redmond, USA) spreadsheet. The 52 assays used were ordered in columns according to their positions on the *Brassica rapa* WGS contigs and/or sequenced BACs. All scores that were determined with the 52 assays were recorded in the spreadsheet and colour-coding was used to differentiate scores that could be ascribed to the A and C subgenomes. Based on this compilation the BACs were selected for validation studies.

In order to determine which of the putative BACs truly mapped to the regions of interest, PCR amplicons (Additional file 2) that spanned the different SNP assays were used. To validate the assignment of BACs to the two subgenomes, PCR products derived from at least two BACs each ascribed to the A and C subgenomes, respectively were subjected to sequence analysis, if available. The resulting sequences were edited, aligned with BioEdit [47] and inspected for SNPs and indels in the region spanning the SNP assays and beyond.

## Electronic supplementary material

Additional file 1: **Sequence information for oligonucleotide pool assays. Bold font indicates sequences specific for a particular oligonucleotide class.** Illumicode sequences are shown in italics. Plain font corresponds to *Brassica* sequences. Spacer nucleotides between the Illumicode sequences and the *Brassica* sequences are underlined. (XLSX 27 KB)

Additional file 2: **Amplicons.** The table lists all amplicons which were used to analyse putative BAC clones for the presence of the homoeologous regions of interest by PCR. (XLSX 25 KB)
